# Double Sequential Defibrillation: An Update of Contemporary Defibrillation Strategies for Pulseless Ventricular Tachycardia and Fibrillation

**DOI:** 10.7759/cureus.77316

**Published:** 2025-01-12

**Authors:** Bryan White

**Affiliations:** 1 Cardiology, Adventist Health System, Rockville, USA

**Keywords:** cpr training, defibrillation vector, double sequential defibrillation, dual sequential defibrillation, out-of-hospital cpr

## Abstract

In a clinical trial, the role of double sequential external defibrillation (DSED) in treating refractory ventricular fibrillation (VF) was evaluated. Many Americans suffer from sudden cardiac arrest daily, and resuscitation rates are low especially in patients with refractory ventricular tachycardia (VT)/fibrillation. This trial was a prehospital resuscitation trial exploring methods to improve meaningful survival in this difficult patient cohort. I applaud the efforts of this trial, along with all of those involved in research and education, in trying to improve outcomes by improving recognition and bystander CPR, reducing delays in EMS activation, initiating CPR and the first delivery of a defibrillation shock, and adjusting pad placement to more successfully defibrillate the patient. In this editorial, I discuss in detail the outcomes of this landmark clinical trial, along with supplemental information submitted by the primary authors. Also, I review the updated defibrillation outcomes by defibrillator pad placement and defibrillation vectors. Finally, I give my recommendation to not include DSED in contemporary resuscitation guidelines and my rationale and justification for my recommendation.

## Editorial

In the Double Sequential External Defibrillation for Refractory Ventricular Fibrillation (DOSE VF) clinical trial, the role of double sequential external defibrillation (DSED) in treating refractory ventricular fibrillation (VF) was evaluated. Four minutes separated the first failed defibrillator shock from the third failed defibrillator shock [1]. This four-minute window can mean the difference between hope and a 3%-15% chance of survival [2-4].

Traditionally, there is heterogeneity in the use and technique of double sequential defibrillation/double external defibrillation. A 2020 systematic review did not find any benefit in key primary outcomes such as survival to hospital discharge, neurological outcome, or the termination of VF/pulseless ventricular tachycardia (VT). Additional reviews have also not proven a benefit [1,2].

In the DOSE VF trial, the authors used a standardized approach [2]. They defined three failed defibrillation shocks as the determinant of refractory VF/pulseless VT (pVT) and did not confound the trial with resuscitative medications such as epinephrine or amiodarone [2-4].

They also described the technique of double sequential defibrillation and vector change defibrillation, reducing the heterogeneity in how this defibrillation method was applied [2-4].

In their Canadian population, EMS agencies that participated in the clinical trial support approximately 4100 out-of-hospital cardiac arrests (OHCA) per year, with 15% being ventricular fibrillatory/pulse ventricular tachycardia in etiology. During the clinical trial, they enrolled a total of 405 patients, including 152 from the first pilot study. Notably, nearly 68% of the out-of-hospital cardiac arrests were witnessed, but only 58% received bystander CPR before EMS arrival [2-4].

They reported that the termination of ventricular fibrillation occurred in 84% of the DSED group versus 68% in the control group of standard defibrillations, with the return of spontaneous circulation (ROSC) occurring in 46% of the DSED group versus 27% in the standard group. Additionally, survival with a good neurological outcome, defined using the modified Rankin Scale score of 2 or less, occurred in 27% of the DSED group versus only 11% in the standard defibrillation group. The vector change did not significantly improve outcomes [2-4].

While the authors’ efforts are commendable, there are several concerns. Prior reviews did not consistently show a benefit to double sequential external defibrillation (DSED). The study was also stopped early due to the impacts of COVID-19 on paramedic staffing and participation, potentially biasing the results. The authors’ power analysis determined that they would need 930 patients, but only 405 were included, with 152 from the original pilot trial [2-4].

Importantly, in the study’s supplementary table, the relative risk for survival to hospital discharge was 1.38 but with a confidence interval ranging from 0.9 to 2.11 [2]. While the intention-to-treat and per-protocol analyses were more promising, the actual treatment received was not as convincing. An effective clinical intervention should be successful in the actual treatment received to ensure successful outcomes in both per-protocol and intention-to-treat analyses, where patient crossover is expected. Since DSED did not show a statistically significant improvement in the actual treatment received, the findings are more likely to be by chance. We should not make guideline-based decisions based on a single trial; the findings are hypothesis-generating but not strong enough to change guideline recommendations [2-4].

A later publication by the same author group revealed that the ideal timing within this trial was a defibrillation separation of less than 75 ms. This suggests the use of two defibrillators to simultaneously defibrillate to achieve the adequate termination of underlying malignant electrical activity that could not be achieved by single defibrillation alone [2-4].

A more recent publication by Lupton et al. demonstrated that in 255 patients with out-of-hospital cardiac arrest (OHCA), an anterior-posterior (AP) pad placement was more successful than an anterolateral (AL) pad placement. Patients with an AP pad placement had a 2.64-fold increase in the adjusted odds ratio of ROSC. In a subgroup analysis of cardiac-only etiologies, the benefit was more pronounced with an AP versus an AL pad placement, extending to survival to discharge and favorable neurological outcomes [5].

Many issues limit effective defibrillation, such as challenges in properly placing defibrillator pads during an emergency due to factors such as obesity, breast tissue, hyperinflated lungs, hair, and diaphoretic skin. In an editorial, Cheskes et al. discussed the role that ventricular hypertrophy or dilatation may have in making defibrillation more difficult [2]. They commented that the combination of two sets of pads may provide a more homogenous distribution of current and that their analyses showed a consistent 10%-20% reduction in impedance. They also found that a higher dose strategy was more effective than a true double sequential approach. The most recent article suggests that a primary AP strategy would be preferable [2-5].

I am encouraged by the work of Cheskes et al. and believe that they are taking steps toward improving survival in patients with cardiopulmonary arrests due to ventricular tachyarrhythmias [2]. I encourage ongoing collaboration between medical device manufacturers and research experts to continue improving defibrillation strategies and developing novel techniques to stop ventricular arrhythmias. There is not enough evidence to mandate AP positioning, vector-changing strategies, and DSED in guidelines, but these findings should lead to ongoing research, clinical trials, and registry data to help shape future recommendations [2-5].

Finally, I want to continue fostering community awareness about the chains of survival, such as the early recognition of sudden cardiac arrest, access to emergency medical care, early CPR, and early defibrillation. Communities should work on their emergency action plans and community education, especially when only 60% of cardiopulmonary arrest victims receive bystander CPR.
